# Immediate Root Filling

**Published:** 1888-04

**Authors:** H. A. Smith

**Affiliations:** Cincinnati, Ohio.


ARTICLE II.

### IMMEDIATE ROOT FILLING.

BY H. A. SMITH, D. D. S., CINCINNATI, OHIO.

[Read before the Mississippi Valley Dental Association, held at Cincinnati, Ohio, March 7, 1888.]

If we were called upon to fill root canals only in teeth from which healthy pulps had been removed by surgical means, I presume the system of "Immediate Root Filling" would be generally adopted. Cases presenting this favorable condition are, however, comparatively rare, and the dentist finds pulps in all stages of inflammation, decomposing and dead pulps, complicated with disease of the peridental membrane and alveolar abscess, either acute or chronic. It is due to this variety in the cases presented, that practitioners necessarily differ in their methods of treatment and in what they regard as the proper time for permanently stopping a root. If there were no other reason for these differences in practice, they would naturally result from temperamental differences in dentists themselves. The late Prof. James Taylor, when speaking of this subject, would say, that we have a class of good practitioners who are forever treating a pulpless tooth; they appear to recognize favorable conditions, but for some reason have not the courage to fill the root canal permanently, fearing possible subsequent trouble.

The typical immediate root filler of our day certainly shows no timidity in this direction; indeed, his courage seems oftener to run away with his judgment. He is ambitious not only to annihilate space, but time also.

The most favorable cases are those in which the pulp has been removed by surgical means, leaving at the apex of the root simply an incised wound, which will heal by first intention; and in this condition, if proper aseptic precautions have been observed in the removal of the pulp, we may fill the canal immediately, with little or no danger of irritation occurring in the apical space. When arsenious acid has been used and the irritant effects of the arsenic have been confined to the pulp, as shown usually by the slight pain attending its removal, we may also, with comparative safety, proceed to fill at once. If, however, in taking away the pulp any considerable hemorrhage follows, that is not arrested spontaneously, it is not best to fill at the same sitting. The application of carbolic acid or the usual styptics to hasten coagulation, will cause more or less injury to the tissues about the apex, including irritation. The better practice in such cases is to leave the clot that has been formed, in contact with the wound until healing has commenced. The blood clot is nature's own dressing and completely filling as it does the root canal, effectually prevents infection from without. By placing an antiseptic cotton dressing in the canal, in contact with the clot, decomposition of the blood will be prevented for several days. In from twenty-four to thirty-six hours, the clot may be removed without disturbing the layer of granular tissue which has formed, and since an antiseptic condition of the root has been maintained, we may, after careful washing proceed to fill permanently. Ordinarily in chronic cases of alveolar abscess with fistula, where systemic conditions are favorable, the root may be filled permanently after thorough cleansing of the pulp cavity and sinus with peroxide of hydrogen, followed by a mild antiseptic such as a dilute solution of carbolic acid or bichloride of mercury.

It is in the management of those cases in which the pulp, having been gangrenous, and as a source of infection for a long period, has caused abscess, with discharge either through the root canal or alveolus, that a radical difference in practice prevails between the conservative practitioner and the advocate of immediate root filling. The statement that all roots can be made aseptic by treatment at one sitting we cannot accept. We have all seen how readily the dentine and cementum of roots of teeth take up coloring matter from the old-fashioned copper amalgam with which they have been filled. If this coloring matter will thus penetrate these dense tissues, certainly septic matter from a pulp that has long been gangrenous will thoroughly infiltrate the tissues of the root, often reaching the peridental membrane. We can then hope to counteract sepsis in these cases only by repeated washings with disinfectants, followed each time with an antiseptic which will not combine with organic matter in such a way as to prevent the effects of subsequent treatment. When we have decided to fill the root, the use of a powerful coagulator, such as chloride of zinc, as a last dressing is indicated. This "cooks" or "vulcanizes" the albuminous matter and renders it, as it were, fixed material. When oxychloride of zinc is used to fill the root, this effect is produced by the zinc chloride in the material.

The attempt to obtain an aseptic condition by burring out the root canal in which the pulp has been in a putrid condition for some time, would it appears be idle; as would also the practice of correcting sepsis by introducing into the root canal the point of a heated instrument.

Those who advocate immediate root filling it appears place great reliance upon what is termed, "vis medicatrix naturæ." This recuperative vitality is, in the mouth, as in all parts of the body, an unknown factor, and cannot be measured accurately. Since the source of contagion in these chronic cases is not confined to the pulp chamber, we would do well to keep the root canals open until we have some better evidence of cure than the cessation of discharge through the external opening, which may follow a single treatment. Granulation tissue may form in the apical space, and along the walls of the sinus and the fistula may heal; yet a cure is not certainly effected. The balance between health and disease which obtained for a short time, has turned on the side of disease; the new tissue formation gives way and we still have a chronic case of abscess. May we not treat such an abscess through the opening in the gum after having filled the root? We may; but not nearly so effectually as when the opening is maintained through the root.

In cases of blind abscess and other trouble following this hasty method of treatment, the surgical procedure of drilling down upon the abscess is freely spoken of as an effective and easy operation. In all surgical treatment two people are to be considered, the operator and the one operated upon. The immediate root filler in the zeal of his universal method, seems to have left out of consideration the person upon whom he inflicts the wound. Besides the uncertainty of affording relief by drilling through the alveolus in cases of forming abscess, is the disagreeable fact that the operation should be performed at the most painful stage in the inflammation, that is, just before the formation of pus.

The papers and discussions which have appeared on this subject in the last two years have, at least, had a wholesome effect upon our daily practice. It has resulted in the adoption of better methods of antisepsis and a stricter cleanliness of both the operator's instruments and person; the action of certain medicaments has become better known, and the relative potency of a great number of antiseptics carefully studied. More exact methods of root filling are being practiced, and we shall, no doubt, in the future have more uniformly good results in our efforts to save pulpless teeth.

DISCUSSION.

Dr. Atkinson spoke of the septic condition of roots and the necessity of cleaning to, and ofted beyond the apex. He stated how uric acid accumulated and the necessity of its speedy elimination from the system.

Regarding immediate root filling, if there is no periostitis he said to go through the foramen and fill immediately, as it is not necessary in most cases to wait any length of time. In alveolar abscess we get suppuration and hyperplasis. After speaking of these and pus formation, he said to relieve the patient from severe pain, while opening up an abscessed tooth, to tie a strong cord to the neck of the tooth and request the patient to pull on this and not loosen the hold. If the pain is still felt, request him to pull a little harder, and keep this up until you have drilled through the foramen; and if you go beyond the apex it will do no harm.

He here spoke about disinfecting being carried to extremes, and that he had years ago used bichloride of mercury for disinfecting in the same manner as that of the present day, for all it is now used *scientifically*.

He said he did not go much on micrologists. He used to be one of them himself. A man that has been to camp meeting and been converted six or seven times generally knows what he is talking about.

In regard to arsenic, or arsenious acid, he said it has no affinity for the tissues and does nothing but take the place of the pabulum in the tissues.

Dr. Watt: After all, the point to be gained in practice is for a man to question himself, and if he cannot answer his own questions and understand himself, he is not fit to act.

Regarding the patient you should not be satisfied until you have inquired of his family history. Perhaps not satisfied with this you may inquire into other things such as constitution, habits, etc. If he has excessive habits as smoking, beer drinking, and the like, do not operate immediately. You must know all of these things before operating, if you want to be successful.

Dr. Conrad: I treat these teeth and in almost all cases fill immediately. After examination wash out the cavity with peroxide of hydrogen and go carefully to the end of the root, and when you know it is clean, fill it. When you find a molar with a calcarious deposit in the pulp chamber, you may think there is no opening into the root canal, but there is, and unless you go through this you will be liable to have trouble. When a root is filled to the apex perfectly with a hard substance, you should never have any trouble. My creed is to wash out the cavity thoroughly with warm water and then peroxide of hydrogen. Be cleanly, thorough, and open into every root canal. Never let a cavity go unfilled where there is a bit of pulp tissue, and never let a root go until it is filled to the end.

Dr. Jay: The gentleman says never leave a portion of a root unfilled. I would like to inquire how he serves the molar roots?

Dr. Conrad: I cut through the crown, where I can, or even through the side just so I get into them. Perhaps we experience more trouble to find the opening in the anterior buccal root of the superior molar than any of the others. Where a root is tortuous, you must not drill straight or you will penetrate the side of the root and have trouble. It is best to go about one half or two-thirds of the length of the root, then with a smaller Morey drill continue a little farther, then use Swiss broaches; keeping the canal flooded with peroxide of hydrogen. When this is carefully worked into the root the effervesence causes the infectious material to be thrown out of the root toward the pulp chamber and not through the apical foramen. Where there is a deposit in the chamber, and you cannot find the opening into the canals, place a little of the Herbst obtundent in the tooth and leave it until the next day, and if necessary repeat the operation until this covering is dissolved away and the opening is exposed.

Dr. Sage: Care should be used in the injection of medicaments into the substance of coagulated lymph or cellular tissue, for inflammatory action is liable to be set up. We often force the medicament through the root to the so called pyogenic tract and get irritation and what we call over-treatment. I think it is not necessary to force any of this medicine through the root unless there is a sinus. The root canal should be kept flooded with antiseptics, and more precaution used if there is no fistulous opening. We should consider the question of diathesis of the patient, as there are certain symptoms we may recognize if we are observing. If a person has high cheek bones, is of stout build with a short neck, and has long bones, it points to a scrofulous diathesis. We have other indications of this in persons of a nervous or sanguine temperament, as those with red or sandy hair, light complexion, long slim necks, and flat chests. In these subjects immediate root filling is not advisable.

Dr. Ames: I believe that where we have no acute inflammation at the time, we can thoroughly remove the contents of the canals and fill immediately. In regard to iodide of potassium I have been in the habit of decomposing it by the galvanic current. Place the negative pole against the face of the patient, or let him hold it in the hand, and place the positive pole on the tissues where you require the decomposition of this substance. My method of disinfecting root canals is to take a Swiss broach, gold plate the point to prevent oxidation, attach to or hold in contact with the positive pole of the battery, and work it carefully and slowly to the apex of the canal, decomposing the iodide and thoroughly disinfecting. Then cleanse and fill the root immediately.

Dr. Jay: I have found nothing better than gutta-percha as a filling material for root canals.

Dr. Taft: There is wonderful unanimity in all these discussions. Every one has said he thinks that roots may be filled as soon as they are ready to be filled. This cannot be denied. We have a great variety of cases presented to us, and we must, as Drs. Watt and Atkinson have stated, take the accidental changes into account. If a patient presents himself and the surrounding circumstances are favorable, you can fill at once; but if a portion of pulp tissue or decomposed matter remain, it is not advisable to do so. Test this by placing a small pledget of cotton into the cavity and leave it for several minutes, then remove. If the cotton smell offensive, and you close up the cavity, you will be liable to have trouble. If there is diseased tissue that needs to be disposed of at the end of the root, break ft up. If this is not necessary, get the root into the best possible condition and seal with chlora percha, being careful to avoid pressure. Care should be exercised in using carbolic acid. It is used too much. Apply the dam and use hot air as a coagulator. All these and other things should be taken into account before filling.

Dr. Fletcher had a failure to report. A man who had taken whiskey, and perhaps quinine, had a superior right lateral that had been sore for five years but had given no particular pain. It needed refilling, and in taking out the filling he found the pulp chamber empty. There was a slight odor and soreness. He used peroxide of hydrogen, as Dr. Conrad suggested, carefully filled the tooth, and dismissed him. The next day he returned suffering from the effects. He punctured the alveolus, got a discharge of blood which relieved the patient somewhat, but to-day that man is in bed.

Dr. Sage spoke of peroxide of hydrogen, its action, etc. He then referred to the treatment of root canals with chloride of zinc to coagulate the albuminous materials.

Dr. Atkinson: When treated in this way, hydro.-chlor.-zincate-of-albumen is formed. On removing this from canals, I have found it to be clear as glass, and have said that I did not know of a more perfect filling, but how long it would remain in this condition I do not know. He spoke of gaseous bodies penetrating the tissues, and that he had seen blood abscesses which required a long treatment and gave all the symptoms of necrosis. Such cases usually succumb to a solution of salt and water followed by applications of aromatic sulphuric acid. In treating diseases where the system needs toning up, he had found McKesson & Robbin's preparation of nux-vomica and phosphorus cantharades of value. Take a two-grain capsule of sulphate of cinchonidia twice a day, and one pill of the nux-vomica preparation at night. This gives the system tone, and then your other medicines will take hold.

When a pus abscess is treated with peroxide of hydrogen, we get a creamy mixture and effervesence. You can prove the action by applying a heated instrument. If carbonic acid is generated, the instrument is colored black; if oxygen is present, the heat is increased; and if you get a flame, it indicates the presence of hydrogen.—*Ohio Journal of Dental Science.*

---

ARTICLE III.

